# The impact of hypothyroidism on the risk of intrahepatic cholestasis of pregnancy: a large-scale study based on pregnant women with hypothyroidism in Shanghai, China

**DOI:** 10.7189/jogh.16.04013

**Published:** 2026-01-12

**Authors:** Mu Lv, Zhijuan Cao, Chuanlu Xu, Xiaoxian Qu, Yirong Bao, Ling Yuan, Hao Ying

**Affiliations:** Department of Obstetrics, Shanghai Key Laboratory of Maternal Fetal Medicine, Shanghai Institute of Maternal-Fetal Medicine and Gynecologic Oncology, Shanghai First Maternity and Infant Hospital, School of Medicine, Tongji University, Shanghai, China

## Abstract

**Background:**

The relationships among clinical hypothyroidism (CH), subclinical hypothyroidism (SCH), and intrahepatic cholestasis in pregnancy (ICP) remain unclear. We aimed to determine the relationship between hypothyroidism and the risk for ICP.

**Methods:**

We conducted this retrospective cohort study at a tertiary care hospital. We used logistic regression analysis to study the risk of ICP, and restricted cubic splines to clarify the quantitative relationship between thyrotropin (TSH) or free thyroxine (FT4) and ICP. We used the Kaplan-Meier method and Cox regression to evaluate the relationship between hypothyroidism and the onset of ICP. Lastly, we checked the Cox proportional hazards assumption using the Schoenfeld residual test.

**Results:**

We included 42 615 pregnant women in the final study. The risk of ICP was higher in the CH group (adjusted odds ratio (aOR) = 3.03; 95% confidence interval (CI) = 2.00–4.58, *P* < 0.001) than in the euthyroidism group. Thyroid peroxidase antibody (TPOAb)(+) CH was also significantly associated with the risk of ICP (aOR = 3.30; 95% CI = 1.92–5.68, *P* < 0.001). However, SCH was not significantly associated with the risk of ICP. Consistent results were observed in the subgroup analysis of ICP based on onset time and severity. Furthermore, reduced FT4 and elevated TSH levels had a dose-response relationship with ICP. Additionally, ICP occurred earlier in the TPOAb(+) CH subgroup than in other groups (log-rank *P* < 0.001; hazard ratio = 3.50; 95% CI = 2.05–5.98, *P* < 0.001).

**Conclusions:**

We found that CH was significantly associated with a greater risk of ICP. CH, especially TPOAb(+) CH, is associated with a greater risk of both early and severe ICP. Furthermore, the prevalence of ICP increases with increasing TSH and decreasing FT4.

Thyroid diseases are the second most prevalent endocrine disorders that affect women throughout the reproductive period [1]. The prevalence of clinical hypothyroidism (CH) and subclinical hypothyroidism (SCH) increases with advancing age [2,3]. Among women of childbearing age in China, the CH prevalence rate is between 0.77–2.05%, the SCH prevalence rate is between 14.25–15.61%, and the positive rate of thyroid peroxidase antibody (TPOAb) is between 11.43–16.12% [3]. Maternal thyroxine is especially important in early pregnancy since the fetal thyroid gland cannot produce iodothyronines until after ten weeks of gestation [4]. Therefore, throughout the crucial first trimester of development, the fetus depends on maternal thyroid hormones crossing the placenta. Maternal hypothyroidism during pregnancy is associated with negative impacts on pregnancy outcomes, including an elevated risk of fetal loss, preeclampsia, placental abruption, and postpartum haemorrhage [5,6]. Additionally, it adversely affects perinatal outcomes, increasing the risks of premature birth, low birth weight, and neonatal respiratory distress syndrome [7,8]. On the other hand, TPOAb is a significant risk factor for autoimmune thyroid disease and, to some extent, indicates the degree of damage to thyroid tissue [9,10]. TPOAb positivity is one of the common factors contributing to hypothyroidism in women during their reproductive period [11]. Therefore, it is important to consider TPOAb status when investigating thyroid function.

The liver is the gastrointestinal organ most significantly affected by functional thyroid disorders [12]. Indeed, a bidirectional relationship between the liver and the thyroid is evident in both physiological and pathological states. The liver, as a primary target organ for thyroid hormones, plays a crucial role in their metabolism, including the synthesis of thyroxine-binding globulin (TBG), peripheral deiodination, and excretion processes. Thyroid hormones regulate hepatic lipid and carbohydrate metabolism, and thyroid dysfunction can influence the onset and progression of liver diseases. Furthermore, there is substantial evidence suggesting that hypothyroidism may have a direct impact on liver structure and function. For instance, decreased serum triiodothyronine levels have been correlated with severe liver diseases such as viral hepatitis [13], alcoholic liver disease [13], and cirrhosis [14], and are linked to poorer prognoses, including increased mortality rates in patients with type A hepatic encephalopathy [15].

Intrahepatic cholestasis of pregnancy (ICP) is a pregnancy-associated liver disease, characterised by pruritus and elevated total bile acid (TBA) levels [16]. The clinical significance of ICP is linked to an increased risk of adverse pregnancy and perinatal outcomes, such as preterm delivery, meconium staining of amniotic fluid, low Apgar scores, and stillbirth [17]. Although emerging evidence emphasises the complex interaction between thyroid hormones and liver function, the relationship between ICP and CH remains ambiguous. While existing evidence highlights the importance of thyroid hormone status in liver physiology and the potential bidirectional effects on cholestasis and metabolic health, the direct connection between ICP and hypothyroidism requires further investigation. Overall, the liver-thyroid axis plays a crucial role in integrating metabolic and excretory functions, wherein thyroid hormones exert a significant influence on hepatic structure and cholestatic processes. Therefore, to further explore the underlying mechanisms and clinical implications, we aimed to investigate the associations between thyroid function indicators, hypothyroidism, and the occurrence of ICP.

## METHODS

### Study population

We conducted this cohort study at the Shanghai First Maternity and Infant Hospital, Tongji University School of Medicine. All pregnant women who had received regular antenatal care at the hospital from February 2013 to October 2017 and met the inclusion criteria were eligible for retrospective enrollment in the study. All participants underwent routine and chronological tests at the first antenatal visit, including thyroid function tests. We extracted data on maternal demographics, including maternal age, pre-pregnancy body mass index (BMI), gestational age, gravidity, parity, and assisted reproduction from the hospital's standardised electronic medical records system, which is regularly maintained and reviewed for accuracy. We adhered to the STROBE guidelines for reporting observational studies in epidemiology (Table S1 in the **Online Supplementary Document**) [18].

**Figure 1:**
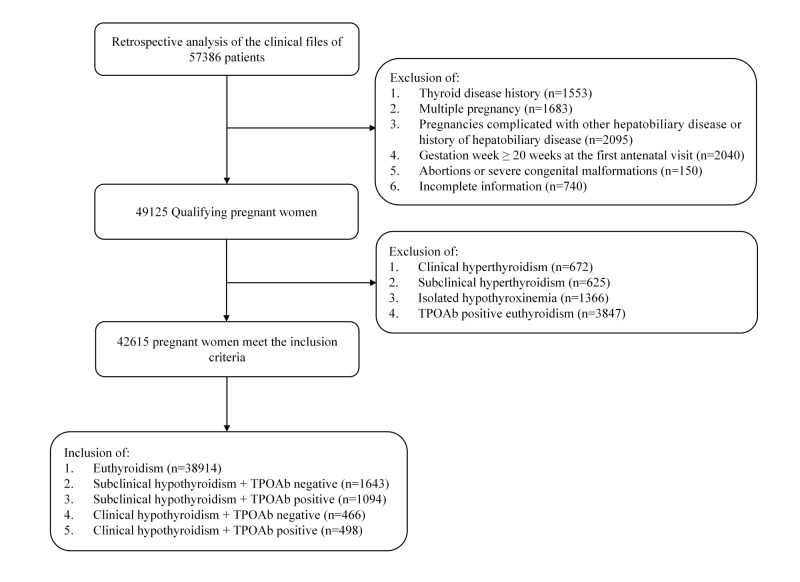
Flowchart of study participants.

We screened 57 386 pregnant women for study inclusion and enrolled 42 615 who met the inclusion criteria (**Figure 1**). The inclusion criteria for study participants were singleton pregnancy and gestation ≤19 + 6 weeks at the first antenatal visit. The exclusion criteria were a history of thyroid disease; clinical hyperthyroidism, subclinical hyperthyroidism, isolated hypothyroxinemia, TPOAb-positive euthyroidism, multiple pregnancies, pregnancy complicated with other hepatobiliary diseases or a history of hepatobiliary disease, gestational week ≥20 weeks at the first antenatal visit, abortion or severe congenital malformation, and incomplete information.

### Thyroid function during pregnancy

The hospital staff collected a maternal blood sample at the first antenatal visit and centrifuged (10-minute with rethawing cycles at 3000 rpm) to obtain the serum. They measured thyrotropin (TSH), free thyroxine (FT4), and TPOAb concentrations using ADVIA Centaur instruments and kits (Siemens, Munich, Germany). Based on our prior institutional studies [19,20], reference intervals for thyroid function were 0.06–3.83 mIU/L for TSH and 1.01–1.57 ng/dL for FT4 in the first trimester, and 0.07–4.08 mIU/L for TSH and 0.95–1.53 ng/dL for FT4 in the second trimester, with TPOAb positively defined at ≥60 IU/mL

We divided the study population into five groups: SCH combined with TPOAb negativity (TPOAb(−) SCH); SCH combined with TPOAb positivity (TPOAb(+) SCH); CH combined with TPOAb negativity (TPOAb(−) CH); CH combined with TPOAb positivity (TPOAb(+) CH); and women with normal thyroid function were defined as euthyroidism.

### Diagnosis of ICP and measurement of outcomes

All cases of ICP were confirmed by the presence of serum TBA above 10 μmol/L, increased liver transaminase enzymes in association with pruritus, and the absence of any other identifiable cause of liver dysfunction [21]. We excluded patients if their pruritus could be attributed to causes other than ICP, or if they had gallstones, cholecystitis, or liver cirrhosis. We extracted ICP diagnoses exclusively from electronic medical records where the diagnosis was formally documented by attending obstetricians or hepatologists. Based on literature definitions, we classified ICP severity by pregnancy peak TBA levels (μmol/L) into the following categories: mild (10–39 μmol/L) and severe (≥40 μmol/L) [22]. According to the time of onset, we divided eligible pregnant women with ICP into an early-onset ICP group (time of initial onset <28 gestational weeks) and a late-onset ICP group (time of initial onset ≥28 gestational weeks) [23]. We aimed to investigate the association between hypothyroidism and the occurrence of ICP. Our primary outcome was the occurrence of overall ICP. The secondary outcomes included the occurrence of early-onset ICP, late-onset ICP, mild ICP, severe ICP, and the time to ICP onset.

### Statistical analysis

We used *R*, version 4.2.2 (R Core Team, Vienna, Austria) for all statistical analyses. We used the Shapiro-Wilk test to assess normality and the Levene test to assess the homogeneity of variances of the quantitative data. We presented variables with significant deviations from normality as medians (interquartile ranges) and normally distributed variables as means (standard deviations). We presented categorical variables as frequencies. We used the Kruskal-Wallis test to compare quantitative factors among the groups, and a χ^2^ test to analyse categorical variables.

We used logistic regression to assess the association between thyroid function and the occurrence of ICP, and to perform ICP subgroup analyses. We performed multivariable logistic regression, adjusting for confounding variables, including maternal age, pre-pregnancy BMI, gestational age, gravidity, parity, and assisted reproduction. We presented the results as odds ratios (ORs) with 95% confidence intervals (CIs). Given the low incidence of ICP, we interpreted the ORs as approximations of relative risks, indicating the magnitude of the association between hypothyroidism and ICP risk. We considered a two-sided *P*-value of <0.05 as statistically significant.

Furthermore, we conducted a subgroup analysis to explore the associations between continuous TSH and FT4 levels and ICP occurrence. We examined the relationships between maternal serum TSH and FT4 levels and ICP using restricted cubic spline curves in logistic regression analyses, with nonlinearities assessed using Wald statistics. The restricted cubic spline curves included five knots at the 5th, 35th, 50th, 65th, and 95th percentiles [24]. To evaluate the relationships between CH, SCH, and the onset time of ICP, we used the Kaplan-Meier method to generate cumulative event curves. We built Cox proportional hazards regression models to evaluate the independent association between hypothyroidism and the primary endpoint. We used the Schoenfeld residual test to verify the proportional hazards assumption in the Cox analysis.

## RESULTS

We included 42 615 cases for analysis after screening for the study eligibility criteria (**Table 1**). Significant differences were observed among the five groups in terms of maternal age, pre-pregnancy BMI, gestational age, gravidity, and parity. However, no significant differences were found in the use of assisted reproduction.

**Table 1:** Comparison of characteristics between patients with euthyroidism and hypothyroidism

	Euthyroidism (n = 38 914)	TPOAb(−) SCH (n = 1643)	TPOAb(+) SCH (n = 1094)	TPOAb(−) CH (n = 466)	TPOAb(+) CH (n = 498)	*P*-value*
**Maternal age†**	30 (28–33)	30 (28–33)	30 (28–33)	30 (28–33)	31 (28–34)	<0.001
**Pre-pregnancy BMI†**	21.44 (19.85–23.39)	21.55 (19.97–23.73)	21.87 (20.08–23.80)	22.17 (20.45–24.65)	22.19 (20.54–24.24)	<0.001
**Gestational week†**	15 (14–16)	15 (14–17)	15 (14–16)	15 (14–16)	15 (14–16)	<0.001
**Gravidity**‡						<0.001
1	21 613 (55.54)	864 (52.59)	567 (51.83)	232 (49.79)	242 (48.59)	
2	10 219 (26.26)	450 (27.39)	304 (27.79)	128 (27.47)	133 (26.71)	
3	4557 (11.71)	191 (11.63)	138 (12.61)	64 (13.73)	63 (12.65)	
≥4	2525 (6.49)	138 (8.39)	85 (7.77)	42 (9.01)	60 (12.05)	
**Parity**‡						0.040
1	30 603 (78.64)	1322 (80.46)	849 (77.61)	358 (76.82)	368 (73.90)	
2	8142 (20.92)	311 (18.93)	238 (21.75)	106 (22.75)	125 (25.10)	
≥3	169 (0.43)	10 (0.61)	7 (0.64)	2 (0.43)	5 (1.00)	
**Assisted reproduction**‡	1156 (2.97)	63 (3.83)	37 (3.38)	10 (2.15)	19 (3.82)	0.145

BMI – body mass index, CH – clinical hypothyroidism, SCH – subclinical hypothyroidism, TPOAb – thyroid peroxidase antibody

*Results for continuous variables are based on the Kruskal-Wallis test; results for categorical variables are based on the χ^2^ test.

†Variables are presented as medians (interquartile range).

‡Variables are presented as numbers (percentages).

The prevalence of ICP was 0.90% for euthyroidism, 0.99% for SCH, and 2.70% for CH pregnant women (**Table 2**). We conducted logistic regression analyses, presenting results from both unadjusted (Model 1) and confounder-adjusted (Model 2; adjusted for maternal age, pre-pregnancy BMI, gestational age, gravidity, parity, and assisted reproduction) models. CH was significantly associated with an elevated ICP risk in both Model 1 (OR = 3.05; 95% CI = 2.04–4.57, *P* < 0.001) and Model 2 (adjusted OR (aOR) = 3.03; 95% CI = 2.00–4.58, *P* < 0.001). Stratifying CH by TPOAb status showed consistent significant associations in both models. For TPOAb(−) CH, Model 1 showed significant results (OR = 2.91; 95% CI = 1.63–5.22, *P* < 0.001), which was confirmed in Model 2 (aOR = 2.74; 95% CI = 1.49–5.04, *P* = 0.001). For TPOAb(+) CH, Model 1 also showed significant results (OR = 3.19; 95% CI = 1.85–5.48, *P* < 0.001), which was also confirmed in Model 2 (aOR = 3.30; 95% CI = 1.92–5.68, *P* < 0.001). However, SCH was not significantly associated with the risk of ICP.

**Table 2:** Logistic regression analysis of the association between hypothyroidism and risk for ICP after stratification by the onset time and severity of ICP

		Model 1*		Model 2†	
	**ICP, n/N (%)**	**OR (95% CI)**	***P*-value**	**aOR (95% CI)**	***P*-value**
**Overall ICP**					
Euthyroidism	350/38 914 (0.90)	ref		ref	
SCH	27/2737 (0.99)	1.10 (0.74–1.63)	0.642	1.09 (0.73–1.62)	0.678
TPOAb(−) SCH	16/1643 (0.97)	1.08 (0.66–1.79)	0.755	1.05 (0.63–1.76)	0.839
TPOAb(+) SCH	11/1094 (1.01)	1.12 (0.61–2.05)	0.715	1.14 (0.62–2.08)	0.673
CH	26/964 (2.70)	3.05 (2.04–4.57)	<0.001	3.03 (2.00–4.58)	<0.001
TPOAb(−) CH	12/466 (2.58)	2.91 (1.63–5.22)	<0.001	2.74 (1.49–5.04)	0.001
TPOAb(+) CH	14/498 (2.81)	3.19 (1.85–5.48)	<0.001	3.30 (1.92–5.68)	<0.001
**Early-onset ICP**					
Euthyroidism	99/38 914 (0.25)	ref		ref	
SCH	9/2737 (0.33)	1.29 (0.65–2.56)	0.461	1.20 (0.60–2.43)	0.606
TPOAb(−) SCH	5/1643 (0.30)	1.20 (0.49–2.94)	0.696	1.04 (0.41–2.66)	0.930
TPOAb(+) SCH	4/1094 (0.37)	1.44 (0.53–3.92)	0.476	1.46 (0.54–3.98)	0.460
CH	7/964 (0.73)	2.87 (1.33–6.19)	0.007	2.48 (1.08–5.68)	0.032
TPOAb(−) CH	3/466 (0.64)	2.54 (0.80–8.04)	0.113	1.70 (0.42–6.93)	0.460
TPOAb(+) CH	4/498 (0.80)	3.18 (1.16–8.66)	0.024	3.20 (1.17–8.76)	0.024
**Late-onset ICP**					
Euthyroidism	251/38 914 (0.65)	ref		ref	
SCH	18/2737 (0.66)	1.02 (0.63–1.65)	0.936	1.02 (0.63–1.66)	0.924
TPOAb(−) SCH	11/1643 (0.67)	1.04 (0.57–1.90)	0.903	1.03 (0.56–1.90)	0.924
TPOAb(+) SCH	7/1094 (0.64)	0.99 (0.47–2.11)	0.983	1.01 (0.48–2.16)	0.972
CH	19/964 (1.97)	3.10 (1.93–4.96)	<0.001	3.23 (2.01–5.18)	<0.001
TPOAb(−) CH	9/466 (1.93)	3.03 (1.55–5.94)	0.001	3.15 (1.61–6.18)	0.001
TPOAb(+) CH	10/498 (2.01)	3.16 (1.67–5.98)	<0.001	3.30 (1.74–6.26)	<0.001
**Mild ICP**					
Euthyroidism	280/38 914 (0.72)	ref		ref	
SCH	20/2737 (0.73)	1.02 (0.64–1.60)	0.947	1.01 (0.64–1.60)	0.968
TPOAb(−) SCH	12/1643 (0.73)	1.02 (0.57–1.81)	0.959	0.99 (0.55–1.78)	0.975
TPOAb(+) SCH	8/1094 (0.73)	1.02 (0.50–2.06)	0.964	1.04 (0.51–2.10)	0.916
CH	17/964 (1.76)	2.48 (1.51–4.06)	<0.001	2.57 (1.57–4.22)	<0.001
TPOAb(−) CH	8/466 (1.72)	2.41 (1.19–4.90)	0.015	2.50 (1.23–5.08)	0.012
TPOAb(+) CH	9/498 (1.81)	2.54 (1.30–4.96)	0.006	2.64 (1.35–5.17)	0.005
**Severe ICP**					
Euthyroidism	70/38 914 (0.18)	ref		ref	
SCH	7/2737 (0.26)	1.42 (0.65–3.10)	0.374	1.38 (0.62–3.04)	0.427
TPOAb(−) SCH	4/1643 (0.24)	1.35 (0.49–3.71)	0.556	1.27 (0.45–3.56)	0.656
TPOAb(+) SCH	3/1094 (0.27)	1.53 (0.48–4.85)	0.474	1.55 (0.49–4.94)	0.457
CH	9/964 (0.93)	5.23 (2.61–10.50)	<0.001	4.77 (2.28–9.99)	<0.001
TPOAb(−) CH	4/466 (0.86)	4.80 (1.75–13.21)	0.002	3.68 (1.15–11.78)	0.028
TPOAb(+) CH	5/498 (1.00)	5.63 (2.26–14.00)	<0.001	5.79 (2.32–14.47)	<0.001

aOR – adjusted odds ratio, CH – clinical hypothyroidism, CI – confidence interval, ICP – intrahepatic cholestasis of pregnancy, SCH – subclinical hypothyroidism, OR – odds ratio, ref – reference, TPOAb – thyroid peroxidase antibody

*Model 1 was unadjusted.

†Model 2 was adjusted for confounding variables, including maternal age, pre-pregnancy BMI, gestational age, gravidity, parity, and assisted reproduction.

To better illustrate the association between thyroid function and the risk of ICP, we performed subgroup analysis. We divided ICP into early-onset and late-onset groups according to the time of initial onset (**Table 2**). We found CH to be significantly associated with an elevated risk of early-onset ICP in both Model 1 (OR = 2.87; 95% CI = 1.33–6.19, *P* = 0.007) and Model 2 (aOR = 2.48; 95% CI = 1.08–5.68, *P* = 0.032). TPOAb(+) CH was also significantly associated with early-onset ICP in both Model 1 (OR = 3.18; 95% CI = 1.16–8.66, *P* = 0.024) and Model 2 (aOR = 3.20; 95% CI = 1.17–8.76, *P* = 0.024). In contrast, there was no strong correlation between SCH and early-onset ICP. CH was strongly associated with late-onset ICP in both Model 1 (OR = 3.10; 95% CI = 1.93–4.96, *P* < 0.001) and Model 2 (aOR = 3.23; 95% CI = 2.01–5.18, *P* < 0.001). Stratification by TPOAb status in CH showed consistent significant associations. For TPOAb(−) CH, Model 1 showed significant results (OR = 3.03; 95% CI = 1.55–5.94, *P* = 0.001), which was confirmed in Model 2 (aOR = 3.15; 95% CI = 1.61–6.18, *P* = 0.001). For TPOAb(+) CH, Model 1 also showed significant results (OR = 3.16; 95% CI = 1.67–5.98, *P* < 0.001), which was also confirmed in Model 2 (aOR = 3.30; 95% CI = 1.74–6.26, *P* < 0.001). Similar to early-onset ICP, SCH subgroups showed no significant associations with late-onset ICP. Overall, the results consistently demonstrated that CH, especially TPOAb(+) CH, was a significant risk factor for both early- and late-onset ICP.

In addition, CH was strongly associated with mild ICP in both Model 1 (OR = 2.48; 95% CI = 1.51–4.06, *P* < 0.001) and Model 2 (aOR = 2.57; 95% CI = 1.57–4.22, *P* < 0.001) (**Table 2**). Stratification by TPOAb status in CH showed consistent significant associations. For TPOAb(−) CH, Model 1 showed significant results (OR = 2.41; 95% CI = 1.19–4.90, *P* = 0.015), which was confirmed in Model 2 (aOR = 2.50; 95% CI = 1.23–5.08, *P* = 0.012). For TPOAb(+) CH, Model 1 also showed significant results (OR = 2.54; 95% CI = 1.30–4.96, *P* = 0.006), which was also confirmed in Model 2 (aOR = 2.64; 95% CI = 1.35–5.17, *P* = 0.005). In contrast, for the overall SCH and SCH subgroups, there was a nonsignificant association with mild ICP. For severe ICP, SCH, whether TPOAb(−) or TPOAb(+), was not significantly associated with severe ICP. However, CH was strongly associated with severe ICP in both Model 1 (OR = 5.23; 95% CI = 2.61–10.50, *P* < 0.001) and Model 2 (aOR = 4.77; 95% CI = 2.28–9.99, *P* < 0.001). Stratification by TPOAb status in CH showed consistent significant associations. For TPOAb(−) CH, Model 1 showed significant results (OR = 4.80; 95% CI = 1.75–13.21, *P* = 0.002), which was confirmed in Model 2 (aOR = 3.68; 95% CI = 1.15–11.78, *P* = 0.028). For TPOAb(+) CH, Model 1 also showed significant results (OR = 5.63; 95% CI = 2.26–14.00, *P* < 0.001), which was also confirmed in Model 2 (aOR = 5.79; 95% CI = 2.32–14.47, *P* < 0.001). The above results indicate that CH, especially when accompanied by positive TPOAb, is significantly associated with an increased risk of mild and severe ICP. In contrast, SCH, regardless of TPOAb status, was not significantly associated with the development of mild or severe ICP.

**Figure 2:**
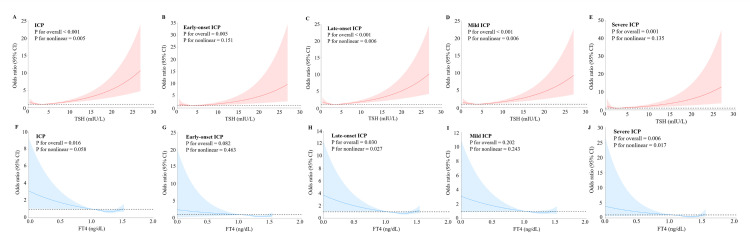
Dose-response relationship between maternal serum TSH and FT4 concentrations and risk of ICP subgroups. **Panel A.** TSH and ICP. **Panel B.** TSH and early-onset ICP. **Panel C.** TSH and late-onset ICP. **Panel D.** TSH and mild ICP. **Panel E.** TSH and severe ICP. **Panel F.** FT4 and ICP. **Panel G.** FT4 and early-onset ICP. **Panel H.** FT4 and late-onset ICP. **Panel I.** FT4 and mild ICP. **Panel J.** FT4 and severe ICP. The models were adjusted for maternal age, pre-pregnancy BMI, gestational age, gravidity, parity, and assisted reproduction. CI – confidence interval, FT4 – free thyroxine, ICP – intrahepatic cholestasis of pregnancy, TSH – thyrotropin.

Restricted cubic spline curves with logistic regression illustrate the dose-response relationship between maternal serum TSH and FT4 levels and ICP, along with its subtypes, using reference values of 3.83 mIU/L for TSH and 1.01 ng/dL for FT4 (**Figure 2****,** Panels A–J). There was a statistically significant nonlinear association between the TSH level and overall ICP (*P* for nonlinear = 0.005), late-onset ICP (*P* for nonlinear = 0.006), and mild ICP (*P* for nonlinear = 0.006). A linear association was observed between the TSH level and early-onset ICP (*P* for nonlinear = 0.151) and severe ICP (*P* for nonlinear = 0.135). In addition, a nonlinear relationship exists between the FT4 level and late-onset ICP (*P* for nonlinear = 0.027) and severe ICP (*P* for nonlinear = 0.017). Conversely, a linear association was observed between the FT4 level and overall ICP (*P* for nonlinear = 0.058), early-onset ICP (*P* for nonlinear = 0.463), and mild ICP (*P* for nonlinear = 0.243).

**Figure 3:**
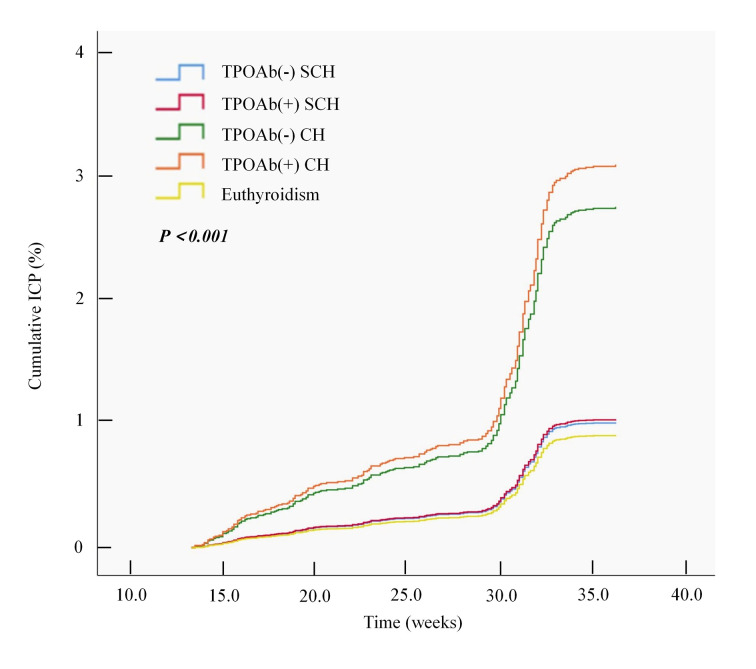
Kaplan-Meier survival curves of the onset time of ICP between patients with euthyroidism and hypothyroidism. CH – clinical hypothyroidism, ICP – intrahepatic cholestasis of pregnancy, SCH – subclinical hypothyroidism, TPOAb – thyroid peroxidase antibody.

The Kaplan-Meier survival analysis curves show the time to ICP onset between the CH and SCH groups (**Figure 3**). The results revealed a significant difference in ICP onset time among the five patient groups (*P* for log-rank test <0.001), with ICP occurring earlier in the TPOAb(+) CH and TPOAb(−) CH groups than in the euthyroidism group. Moreover, multivariate Cox regression analysis revealed that the TPOAb(−) CH (hazard ratio (HR) = 2.86; 95% CI = 1.57–5.22, *P* < 0.001) and TPOAb(+) CH groups (HR = 3.50; 95% CI = 2.05–5.98, *P* < 0.001) were significantly associated with ICP after risk factor adjustment (**Table 3**). We performed the Schoenfeld residual test to verify the proportional hazards assumption in the Cox analysis (Table S2 in the **Online Supplementary Document**). The global test yielded a *P*-value of 0.5168, supporting the validity of the Cox models.

**Table 3:** Multivariate Cox regression analysis for ICP between patients with euthyroidism and hypothyroidism

	Model 1*		Model 2†	
	**HR (95% CI)**	***P-*value**	**HR (95% CI)**	***P*-value**
**Euthyroidism**	ref		ref	
**TPOAb(−) SCH**	1.11 (0.67–1.84)	0.677	1.09 (0.66–1.81)	0.742
**TPOAb(−) SCH**	1.14 (0.63–2.08)	0.668	1.16 (0.64–2.12)	0.624
**TPOAb(−) CH**	3.02 (1.70–5.37)	<0.001	2.86 (1.57–5.22)	<0.001
**TPOAb(−) CH**	3.39 (1.99–5.79)	<0.001	3.50 (2.05–5.98)	<0.001

CH – clinical hypothyroidism, CI – confidence interval, HR – hazard ratio, ICP – intrahepatic cholestasis of pregnancy, SCH – subclinical hypothyroidism, ref – reference, TPOAb – thyroid peroxidase antibody

*Model 1 was unadjusted.

†Model 2 was adjusted for confounding variables, including maternal age, pre-pregnancy BMI, gestational age, gravidity, parity, and assisted reproduction.

## DISCUSSION

We are the first to find an association between hypothyroidism and the risk of ICP. We found that CH was significantly associated with a higher risk of ICP, whether TPOAb-negative or TPOAb-positive, suggesting that CH may be an important risk factor for the development of ICP during pregnancy. In contrast, SCH, regardless of TPOAb status, was not associated with an increased risk of ICP. Furthermore, CH, especially TPOAb(+), was a significant risk factor for both early and severe ICP. There is a statistically significant nonlinear association between TSH and ICP; moreover, a linear association is observed between FT4 and ICP. Accordingly, when formulating and interpreting clinical practice recommendations related to ICP, more attention needs to be given to the care of pregnant women with hypothyroidism.

The risk of ICP was greater for pregnant women with CH than for those with euthyroidism, which indicates that thyroid hormones play an important role in the pathogenesis of ICP. The liver is a typical target organ for thyroid hormones [25], with hepatocytes expressing comparable levels of thyroid hormone receptors (TRα and TRβ) [26]. Hypothyroidism can impair the metabolic and detoxification functions of liver cells, diminish their ability to process bile components, and consequently promote the development of intrahepatic cholestasis [27,28]. Hypothyroidism induces hepatic endoplasmic reticulum stress by reducing mitochondrial oxidative capacity and increasing lipid peroxidation [29]. In pregnancy, endoplasmic reticulum stress in hepatocytes is a known trigger for ICP, as it impairs bile acid synthesis and transport [30], supporting a plausible link between hypothyroid-related hepatocellular stress and ICP development. Beyond hepatic metabolic activities, thyroid hormones also contribute to bilirubin production and composition [31,32]. Hypothyroidism has been associated with cholestatic jaundice in case reports, which is attributed to reduced bilirubin conjugation due to decreased UDP-glucuronyl transferase activity and diminished bile flow resulting from altered canalicular membrane fluidity and increased cholesterol-phospholipid ratios. These changes may affect several canalicular membrane transporters and enzymes, including Na^+^, K+-ATPase [33]. The triad of reduced bilirubin excretion, hypercholesterolemia, and hypotonia of the gallbladder observed in patients with hypothyroidism may impair bile acid efflux transporters [34]. Rodent studies show that hypothyroidism reduces hepatic bile salt export pump BSEP expression, leading to impaired bile acid excretion into bile ducts and subsequent cholestasis [35].

Overexpression of TSH down-regulates sodium-taurocholate cotransporting polypeptide NTCP mRNA levels, further disrupting bile acid uptake and metabolism [36] – findings that align with our observation of increased ICP risk in hypothyroid women. Furthermore, we found that SCH, regardless of TPOAb status, was not associated with an increased risk of ICP. This may be attributed to the fact that SCH represents an early stage of CH, with the essential difference between the two conditions being the degree of thyroid dysfunction and the compensatory ability of thyroid hormones. When thyroid cell damage progresses, and normal thyroid hormone levels cannot be maintained, SCH progresses to CH. Therefore, patients with CH exhibit a greater risk of developing ICP than those with SCH.

Typically, ICP is classified as early-onset or late-onset based on the gestational week of onset and as mild or severe based on severity. There is a certain correlation between the onset time and the ICP severity. Early-onset ICP is often associated with greater severity than late-onset ICP. Early-onset ICP typically manifests as more severe hepatic injury during early pregnancy, accompanied by more profound maternal-fetal bile acid metabolic disorders, which can exacerbate the severity of ICP. [37]. Additionally, early-onset ICP is correlated with an elevated risk of adverse fetal outcomes, including stillbirth and meconium aspiration [38]. We demonstrated that pregnant women with CH had an earlier onset and more severe ICP than normal pregnant women did. Several metabolic genes in the liver, such as malic enzyme, Fas, and Cpt-1α, are directly regulated by the interaction between thyroid hormones and TR [39]. Hypothyroidism may lead to the obstruction of small bile ducts in the liver by lipid deposits, impeding bile excretion and resulting in intrahepatic cholestasis [40,41]. On the other hand, insufficient thyroid hormone levels can impair myocardial contractility, reduce cardiac output, and decelerate systemic blood circulation [42,43]. Blood perfusion to the liver diminishes correspondingly, resulting in hepatic hypoxia. This hypoxic condition can inflict damage on hepatocytes, impair the liver's metabolic and excretory functions, and induce constriction of intrahepatic blood vessels. Such vascular constriction exacerbates bile excretion obstruction, culminating in intrahepatic cholestasis. Therefore, CH drives earlier ICP onset by disrupting thyroid hormone-mediated bile acid homeostasis and enhancing severity through increased cytotoxicity, oxidative stress, and vascular dysfunction.

In our study, TPOAb(+) CH patients had a higher risk of both early-onset ICP and severe ICP than TPOAb(−) CH patients, indicating that TPOAb plays an important role in regulating ICP in hypothyroidism. The reason may be attributed to the complex interplay among thyroid dysfunction, immune-inflammatory responses, bile metabolism disorders, and other interconnected processes. Thyroid autoantibodies can target the thyroid gland, cause thyroid damage, and also affect extrathyroidal tissues, such as the liver, promoting the progression of liver fibrosis in autoimmune thyroid diseases [44]. The presence of TPOAb, indicative of autoimmune thyroid inflammation such as Hashimoto's thyroiditis, affects the liver through various mechanisms. The systemic release of proinflammatory cytokines, including IL-6 and TNF-α, from the inflamed thyroid can induce hepatocyte inflammation and impair bile canalicular function [45]. Primary biliary cholangitis often coexists with autoimmune thyroid disorders, such as Hashimoto’s thyroiditis, where hypothyroidism exacerbates cholestasis by disrupting ABCB4-mediated biliary phospholipid excretion [46,47]. Additionally, TPOAb may cross-react with hepatocyte surface antigens, directly attacking liver cells and impairing bile excretion. Thus, the above mechanisms indicate that positive TPOAb can exacerbate the hepatic impact of hypothyroidism, thereby leading to earlier onset and increased severity of ICP.

Our findings linking early pregnancy CH to increased ICP risk offer concrete guidance to optimise antenatal care for high-risk women. We emphasise that not all thyroid disorders carry the same risk. Our findings show that CH and TPOAb(+) CH are significant risk factors, while SCH is not. Given our finding that ICP occurs earlier in the TPOAb(+) CH subgroup, we recommend that pregnant women with CH, particularly those who are TPOAb-positive, should be considered a high-risk group for ICP and warrant intensified surveillance. For these high-risk patients, we propose educating them about the early signs of ICP and initiating a protocol of more frequent liver function tests and monitoring bile acid levels. In addition, we highlight the dose-response relationship between thyroid hormone levels (reduced FT4, elevated TSH) and ICP risk as a potential tool for dynamic risk assessment. Early identification of CH allows for timely monitoring of ICP and, when indicated, levothyroxine treatment, which may help mitigate thyroid-related metabolic disruptions linked to cholestasis.

The strengths of this study include our combined analysis of the association between hypothyroidism and ICP, as well as our stratification by onset time and ICP severity. These findings have not been reported in previous studies. In addition, our sample size was relatively large compared to prior studies. However, our study has certain limitations. First, we did not consider the use of levothyroxine in pregnant women with hypothyroidism. Second, thyroid autoantibodies include TPOAb, thyroglobulin antibody, and thyroid receptor antibody; however, we only investigated TPOAb. Third, as this is only an epidemiological study, research is lacking into the molecular mechanisms underlying the interaction between hypothyroidism and ICP. In addition, the single-centre, retrospective design is also a limitation of our study, primarily due to the potential for referral bias and uniformity in clinical practice. Our findings require validation in future multi-centre, population-based studies to account for regional variations and minimise referral bias. In our study, we strictly adhered to the inclusion and exclusion criteria and excluded any participant with missing data on variables critical to these criteria. The assessment of thyroid function was based on a single measurement at baseline, which may not fully capture long-term thyroid status and is susceptible to within-individual biological variation. Thyroid function parameters were measured using standardised assays in our hospital’s clinical laboratory, but inter-assay variability could introduce minor measurement error. We sought to minimise this by using standardised protocols and validated diagnostic criteria throughout the study. In addition, our analysis involved several subgroup analyses to explore the robustness of our primary finding. While this approach provides a comprehensive view of the data, it increases the probability of false-positive findings. Therefore, both basic and clinical research are needed to further validate the findings of this study.

## CONCLUSIONS

We found that CH, whether TPOAb-negative or TPOAb-positive, was significantly and independently associated with a greater risk of ICP, suggesting that CH may be an important risk factor for the development of ICP during pregnancy. However, SCH, regardless of TPOAb status, was not associated with an increased risk of ICP. Furthermore, patients with CH, especially TPOAb(+), have a greater risk of both early- and severe ICP. Screening for hypothyroidism during pregnancy may enable clinicians to subclinically predict the occurrence of ICP during pregnancy. However, further research is needed to elucidate the potential mechanisms underlying the relationship between hypothyroidism and ICP.

## Additional material


Online Supplementary Document
